# Posterior staphylomas and scleral curvature in highly myopic children and adolescents investigated by ultra-widefield optical coherence tomography

**DOI:** 10.1371/journal.pone.0218107

**Published:** 2019-06-10

**Authors:** Noriko Tanaka, Kosei Shinohara, Tae Yokoi, Kengo Uramoto, Hiroyuki Takahashi, Yuka Onishi, Shintaro Horie, Takeshi Yoshida, Kyoko Ohno-Matsui

**Affiliations:** 1 Department of Ophthalmology and Visual Science, Tokyo Medical and Dental University, Bunkyo-ku, Tokyo, Japan; 2 Tokyo Metropolitan Hiroo Hospital, Shibuya-ku, Tokyo, Japan; 3 Musashino Red Cross Hospital, Musashino-shi, Tokyo, Japan; Singapore National Eye Centre, SINGAPORE

## Abstract

**Purpose:**

To determine the early signs of posterior staphylomas in highly myopic eyes of younger subjects by swept-source ultra-widefield optical coherence tomography (WF-OCT).

**Methods:**

This was an observational case series study. Highly myopic subjects younger than 20 years old who were examined consecutively by prototype WF-OCT were studied. High myopia was defined according to the Ministry of Health and Welfare, Japan classification. A posterior displacement of the sclera and two OCT features indicating the staphyloma edges were used as markers of a staphyloma.

**Results:**

Fifty-five eyes of 30 patients with the mean age of 12.3 years, and the mean axial length of 27.9 mm were studied. Seven of the 55 eyes (12.7%) had a posterior displacement of the sclera and were diagnosed as having a staphyloma. Among the two OCT features of the staphyloma edges, a gradual thinning of the choroid toward the staphyloma edge and gradual re-thickening of choroid from the staphyloma edge toward the posterior pole were found in these 7 eyes. However, the other feature of an inward protrusion of the sclera at the staphyloma edge, was obvious in only 2 eyes. The subfoveal choroid and choroid nasal to the optic disc were significantly thinner in eyes with a staphyloma than those without it.

**Conclusions:**

The changes of the choroidal thickness toward the staphyloma edge with the posterior displacement of the sclera were considered an early sign which precedes an inward protrusion of sclera at the staphyloma edge.

## Introduction

A posterior staphyloma is a hallmark abnormality of the globe of eyes with pathologic myopia.[1–4] Spaide defined a posterior staphyloma as, "an outpouching of the wall of the eye with a radius of curvature less than the radius of curvature of the surrounding eye wall”.[3] The results of earlier studies showed that highly myopic eyes with a posterior staphyloma had significantly poorer vision and higher frequencies of anatomical anomalies than highly myopic eyes without a staphyloma.[5–7]

In spite of their importance, reliable and objective methods to detect posterior staphylomas has not been available. In earlier studies, conventional 50° stereoscopic fundus photographs and B mode echograms were used to detect the presence of a staphyloma. However, most of the staphylomas were too wide to be recorded in 50° fundus photographs. In addition, it was difficult to detect very early stages of staphylomas by these methods, and staphylomas became detectable only when they became deep enough or when they were accompanied by pigmentary alterations along the staphyloma edges.

As described in detail previously, [7, 8] Shinohara et al. reported on the usefulness of WF-OCT in detecting posterior staphylomas, and they stated that the morphological hallmarks of posterior staphylomas were a smooth border with a gradual thinning of the choroid from the periphery towards the edge of the staphyloma and a gradual re-thickening of the choroid in the direction towards the posterior pole. These findings have also been reported in peripapillary staphylomas and inferior staphylomas in other studies.[9, 10]

Staphylomas are generally considered to develop in later life because the formation of a staphyloma is gradual, and it is difficult to determine when and how it develops. However, it has been reported in earlier studies that staphylomas were observed in younger individuals, although the incidence of a staphylomas in younger individuals varied among these earlier studies.[1, 11–14] The purpose of this study was to determine the early changes of posterior staphylomas in children including teenagers with this new technology of swept-source WF-OCT.

## Materials and methods

The procedures used in this study conformed to the tenets of the Declaration of Helsinki and were approved by the Ethics Committee of Tokyo Medical and Dental University (The approval number: M2000-2278). A signed informed consent was obtained from all the participants and their parents or guardians. This study was performed on highly myopic subjects younger than 20-years-of-age who were consecutively examined by WF-OCT between February and October 2017. High myopia was defined as a myopic refractive error (spherical equivalent) greater than -8.0 diopters (D) for those >8 years and -6.0 diopters (D) for those between 6 and 8 years, or an axial length longer than 26.5 mm, according to the definition of the Ministry of Health and Welfare, Japan.[15] The refractive errors were measured under the pupil dilatation with 0.5% tropicamide/phenylephrine. The exclusion criteria were; patients with poor quality OCT images, a history of vitreoretinal surgery, and systemic or ocular diseases which could cause or are associated with high myopia, e.g., congenital stationary night blindness.

All patients had a comprehensive ophthalmological examination including measurements of the best-corrected visual acuity (BCVA), refractive error, and axial length (IOL Master, Carl Zeiss Meditec, Jena, Germany). Dilated stereoscopic fundus examinations, color fundus photography (TRC-50DX, Topcon, Tokyo, Japan; or Optos 200Tx scanning laser ophthalmoscopy; or Optos PLC, Dunfermline, Scotland, UK) were performed on all eyes.

Ultra-widefield OCT images were obtained with a prototype swept-source WF-OCT instrument (Canon Corp., Tokyo, Japan) with an A-scan repetition rate of 100,000 Hz. The light source was a tunable laser centered at 1050 nm with a 100 nm tuning range. The scanned line length was 23 mm in the horizontal direction and 20 mm in the vertical direction, and the scan depth was 5 mm. Cross-sectional scans and 12 radial scans centered on the fovea were obtained.

To detect signs of staphylomas or changes of the scleral curvature by WF-OCT images, we focused on a posterior displacement of the sclera from the surrounding scleral curvature, and the two OCT features suggesting the staphyloma edges as reported by Shinohara et al.[7, 8] The two representative findings at the staphyloma edges were a gradual thinning of the choroid from the periphery towards the edge of the staphyloma and a gradual re-thickening of the choroid in a direction toward the posterior pole, and an inward protrusion of the sclera at the edge of a staphyloma. The diagnosis of a posterior staphyloma was made when the OCT findings included a posterior displacement of the sclera relative to the surrounding sclera which was essential as the definition of staphyloma, and at least one of the two typical OCT features suggesting the staphyloma edges. These findings were judged to be present when they were observed in more than two consecutive sections in the 12 radial scans. All assessments were made by two retina specialists (KS and KOM).

The staphyloma types were classified according to those reported by Ohno-Matsui.[11] To determine the clinical characteristics of patients with a staphyloma, the following factors were determined; presence of myopic maculopathy lesions according to META-PM classification, [16] the choroidal and retinal thicknesses, incidence of peripapillary diffuse chorioretinal atrophy (PDCA). The presence of myopic maculopathy lesions and the PDCA were based on their presence in the color fundus photographs. The horizontal and vertical OCT sections across the fovea were used for the measurements of the choroidal and retinal thickness. The retinal thickness was measured as the distance between the internal limiting membrane and the outer surface of the neurosensory retina. The choroidal thickness was defined as the distance between the outer border of the retinal pigment epithelium-Bruch’s membrane complex and the chorioscleral border. The retinal thickness measurements were made at the fovea, and the choroidal thickness measurements were made at the fovea, 2500 μm nasal to the fovea, 2500 μm temporal to the fovea, 2500 μm superior to the fovea, and 2500 μm inferior to the fovea. Because a staphyloma was expected to include the area nasal to the optic disc in some cases, we also measured the choroidal thickness at 2000 μm nasal to the optic disc. The measurements of the retinal and choroidal thickness were performed by one masked examiner (NT).

### Statistical analyses

The SPSS 22.0 software (IBM-SPSS, Inc., Chicago, IL, USA) was used for all statistical analyses. We compared the eyes with or without a staphyloma in the BCVA, refractive error, axial length, and the retinal and choroidal thickness using the Mann-Whitney *U* tests. Also, we compared the incidence of myopic macular lesions more severe than the diffuse chorioretinal atrophy (category 2) which was equivalent to the definition of the pathologic myopia and PDCA between eyes with and without a staphyloma. The significance of the differences in the incidences was determined by Chi-square tests or Fishers exact tests. A *P*-value <0.05 was considered statistically significant.

## Results

The study included 56 eyes of 30 patients. One eye was excluded due to the suspect of congenital stationary night blindness. No eyes were excluded due to the poor quality. Eventually 55 eyes of 30 consecutive patients were studied. The mean age was 12.3 ± 4.0 years with a range of 6 to 19 years. The mean refractive error (spherical equivalent) was -10.8 ± 2.9 diopters (D) with a range of -17.5 to -6.8 D. The mean axial length was 27.9 ± 1.6 mm with a range of 25.0 to 32.6 mm.

**Fig 1:**
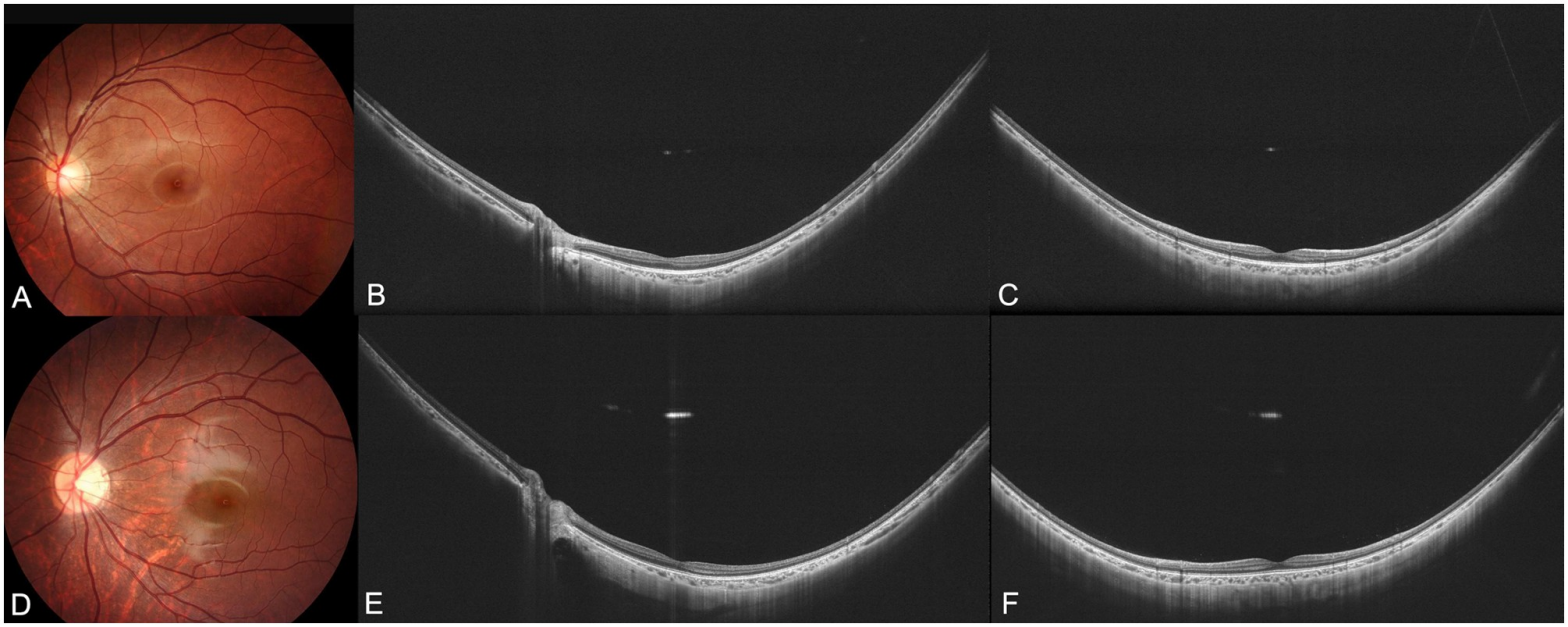
Images of highly myopic eyes without an evident staphyloma. (A)-(C) Left eye of a 16-year-old young man with an axial length of 27.6 mm. (A) Left fundus photograph showing an almost normal fundus. (B) Horizontal widefield optical coherence tomographic (WF-OCT) image showing a uniform thickness of the choroid. The inner scleral surface shows a smooth arc. (C) Vertical WF-OCT section showing a smooth arc of the inner scleral surface. Choroid appears to be thinner toward the periphery, however the transition of the choroidal thickness is gradual and smooth. (D)-(F) Images of the left eye of an 8-year-old boy with an axial length of 26.0 mm. (D) Fundus photograph shows slight tessellation only temporal to the optic disc. (E) Horizontal WF-OCT image showing a uniform thickness of the choroid although the inner scleral surface has an asymmetric arc. Inner scleral surface has a smooth arc. (F) Vertical WF-OCT section showing a smooth arc of the inner scleral surface. Choroid appears to be thinner toward the periphery, however the transition of choroidal thickness is gradual and smooth.

**Fig 2:**
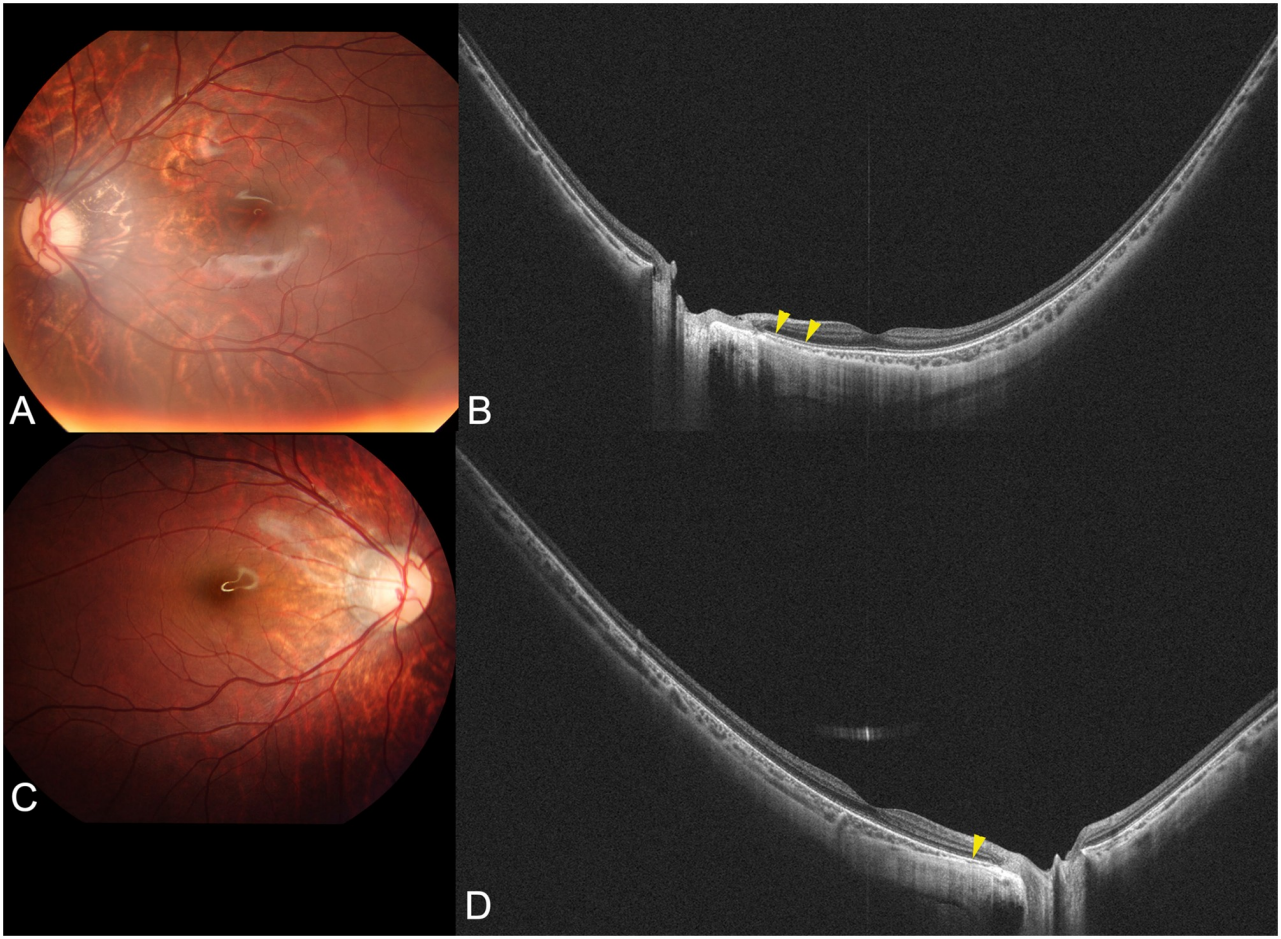
Images of highly myopic eyes with peripapillary diffuse atrophy but without a posterior staphyloma. (A) Fundus photograph of the left eye of a 9-year-old boy with an axial length of 28.4 mm showing marked tessellation as well as peripapillary diffuse chorioretinal atrophy (PDCA) temporal to the optic disc. (B) Horizontal WF-OCT image shows a marked thinning of the choroid (arrowheads) in the area of the PDCA. The transition of surrounding choroid to almost absent choroid in the area of the PDCA is sudden. However, the inner scleral curvature is smooth and no posterior dislocation is seen. (C) Right fundus photograph of an 8-year-old boy with an axial length of 26.0 mm showing peripapillary diffuse atrophy temporal to the optic disc. (D) Horizontal WF-OCT image showing a marked thinning of the choroid (arrowhead) in the area of the PDCA. The transition of the surrounding choroid to almost absent choroid in the area of the PDCA is sudden. However, the inner scleral curvature is a smooth arc and no posterior dislocation is seen.

**Fig 3:**
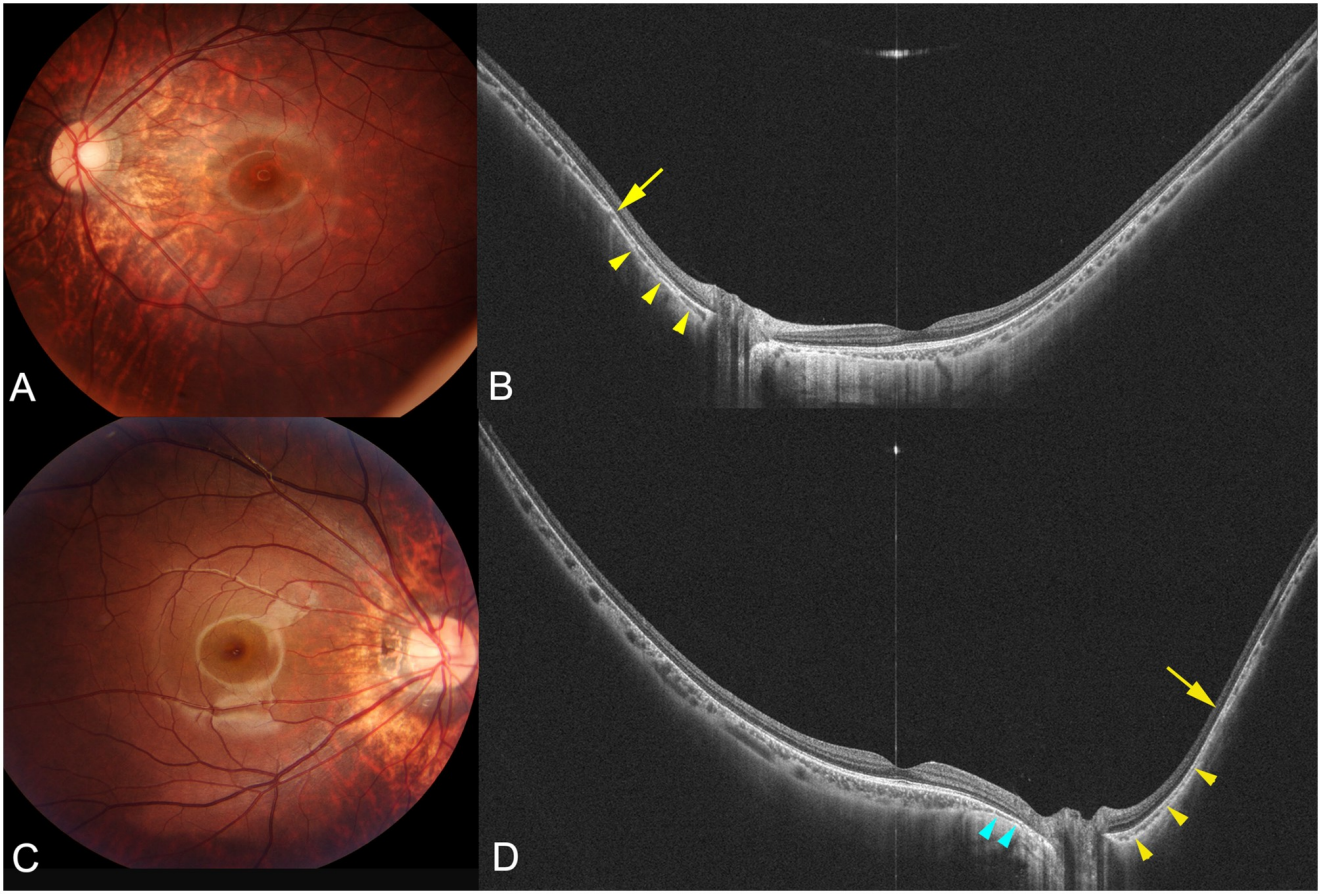
WF-OCT images of highly myopic eyes with peripapillary staphyloma without a scleral inward protrusion. (A) Fundus photograph of the left eye of an 11-year-old girl with an axial length of 27.1 mm showing peripapillary diffuse atrophy. (B) Horizontal WF-OCT image shows that the nasal choroid gradually thins toward the staphyloma edge (arrow) and gradually re-thickens toward the posterior pole (arrowheads). Inner scleral surface is posteriorly displaced in the area between the edge of the staphyloma (arrow) and nasal edge of the optic disc, compared to the curvature more nasal to the staphyloma edge. However, the scleral inward protrusion at staphyloma edge is not obvious. (C) Right fundus photograph of a 17-year-old young man with an axial length of 28.1 mm showing peripapillary diffuse chorioretinal atrophy (PDCA). (D) Horizontal WF-OCT image showing that the inner scleral surface is slightly displaced posteriorly nasal to the optic nerve. The choroid gradually thins toward the edge of the staphyloma (arrow) and re-thickens toward the posterior pole (yellow arrowheads). However, scleral inward protrusion at the staphyloma edge is not obvious. Choroidal thickening closer to the optic nerve appears to be similar to peripapillary intrachoroidal cavitation. In the area of the PDCA (blue arrowheads), the scleral curvature is also displaced posteriorly.

**Fig 4:**
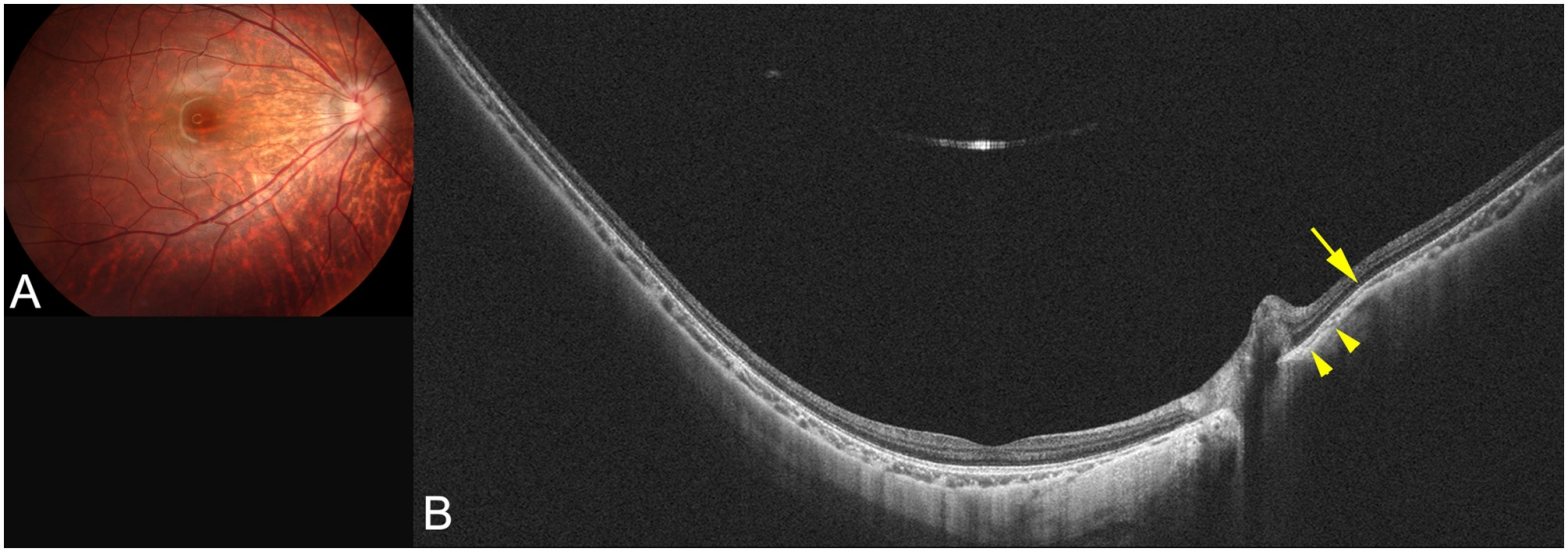
WF-OCT image of a highly myopic eye with peripapillary staphyloma and a scleral inward protrusion. (A) Fundus photograph of the right eye of an 11-year-old girl with an axial length of 26.8 mm showing peripapillary diffuse chorioretinal atrophy (PDCA). (B) Horizontal WF-OCT image showing a posterior displacement of the inner scleral surface nasal to the optic disc compared to the surrounding curve. The choroid at the edge of the staphyloma gradually thins toward the staphyloma edge (arrow) and gradually re-thickening toward the posterior pole (arrowheads). Scleral inward protrusion is also seen at the staphyloma edge (arrow).

**Fig 5:**
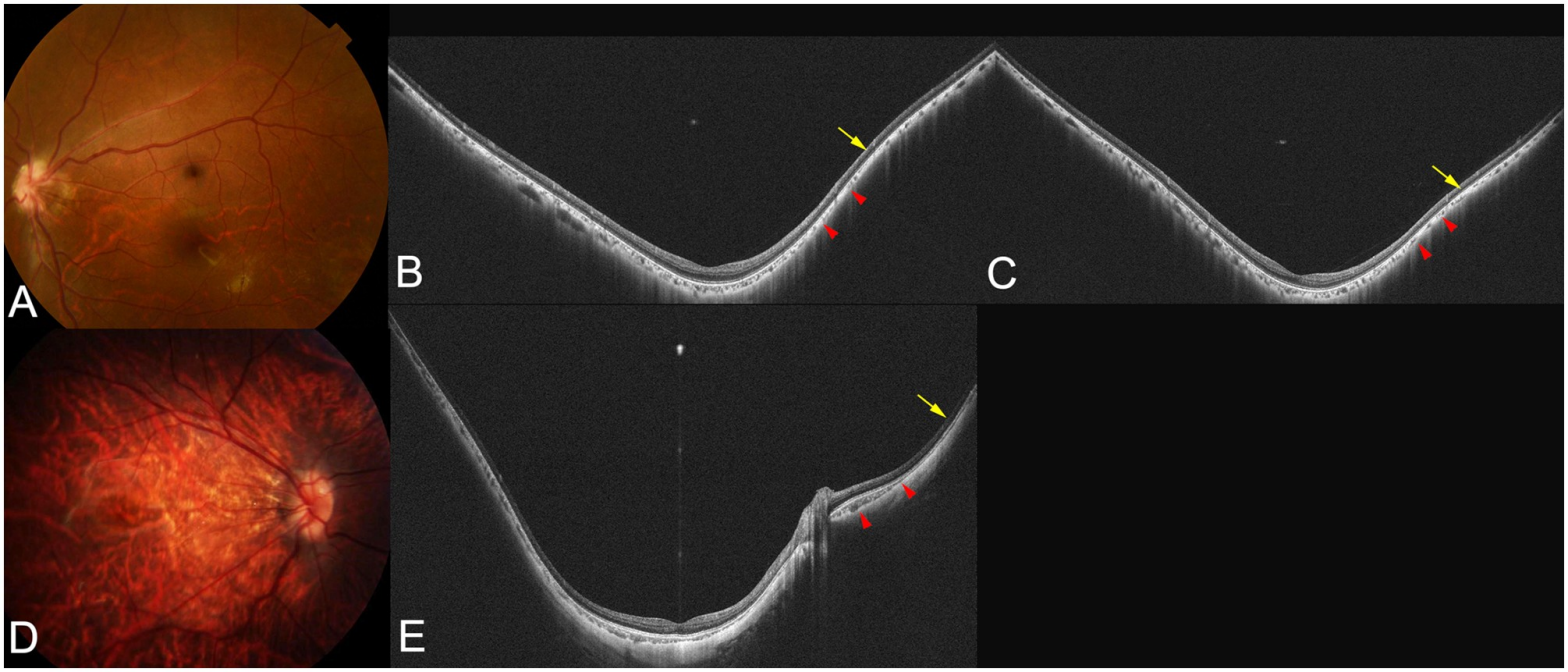
Images of highly myopic eyes with macular staphyloma. (A) Fundus photograph of the left eye of an 18-year-old young woman with an axial length of 27.0 mm showing peripapillary diffuse chorioretinal atrophy (PDCA). (B) Vertical WF-OCT section across the fovea shows a slight elevation of the inner scleral curvature indicating the edge of the staphyloma (arrow). The sclera is posteriorly dislocated more central from the staphyloma edge. The choroid gradually thins toward the edge of the staphyloma and gradually re-thickens toward the posterior pole. The inner sclera is slightly displaced posteriorly (arrowheads) in the area with re-thickening of choroid. (C) In the adjacent OCT section, the same features of the staphyloma edge (scleral inward protrusion and choroidal thinning and re-thickening) are seen. The inner sclera is slightly dislocated posteriorly (arrowheads) in the area with re-thickening of choroid. (D) Fundus photograph of the right eye of an 18-year-old young man with an axial length of 30.8 mm showing diffuse chorioretinal atrophy and multiple lacquer cracks. (E) Nasal choroid gradually thins toward the staphyloma edge and gradually re-thickens toward the optic nerve. Inward scleral protrusion at the edge of the staphyloma is not obvious. However, the sclera is posteriorly displaced in the area between staphyloma edge and the nasal edge of optic nerve. The macular sclera is also dislocated posteriorly, however no obvious OCT features of a staphyloma edge is seen temporal to the fovea.

Among the 55 eyes, 7 eyes (12.7%) were diagnosed as having a staphyloma. WF-OCT images of highly myopic eyes without an evident staphyloma are shown in Figs 1 and 2, and those with a staphyloma are shown in Figs 3–5. In the 48 highly myopic eyes without a staphyloma, the WF-OCT images showed that the curvature of inner surface of the sclera had a smooth arc without any elevations or depressions (Fig 1). The choroidal thickness appeared to be somewhat variable depending on the area, and in most cases the choroid was the thinnest temporal to the optic disc. However, the transition of choroidal thickness was smooth and gentle in 24 of the 48 eyes (50.0%). The remaining 24 of the 48 eyes (50.0%) had a sudden and marked thinning of the choroid temporal to the optic disc (Fig 2). This finding was identical to what had been reported to be peripapillary diffuse chorioretinal atrophy (PDCA), [17, 18] which is a funduscopic feature of children that have a future progression to pathologic myopia as adults. However, in these 24 eyes, a scleral posterior displacement was not found (Fig 2).

In contrast, in the 7 eyes with a staphyloma, a posterior displacement of the sclera was observed (Figs 3–5). The type of staphyloma was the peripapillary type in 4 eyes and the wide macular type in the remaining 3 eyes. Among the two OCT features of staphyloma edge, the gradual thinning of the choroid toward the staphyloma edge and gradual re-thickening of choroid from the staphyloma edge toward the posterior pole, was found in all the 7 eyes. However, the other OCT feature of the staphyloma edge, i.e., a scleral inward protrusion, was detected in only 2 eyes (Fig 4). Both of these eyes had a peripapillary staphyloma and a scleral inward protrusion was prominent nasal to the optic disc. In the other 5 eyes with a staphyloma, the sclera was dislocated posteriorly in the staphylomatous area, however the inward scleral protrusion at the staphyloma edge was not obvious. In the 2 of the 4 eyes with a peripapillary staphyloma, the choroid appeared to be thickened as it approached the optic nerve (Fig 3).

The characteristics of eyes with and without a staphyloma are shown in Table 1. Statistical comparisons showed that the differences in the age, BCVA, refractive error, and axial lengths between the two groups were not significant. All the 7 eyes with an evident staphyloma had myopic maculopathy lesions of category 2 (diffuse atrophy). None of the eyes had category 0 (no myopic maculopathy lesion) or category 1 (tessellated fundus). In 48 eyes without an evident staphyloma, 17 eyes (35.4%) had category 1 (tessellated fundus), 24 eyes (50.0%) had category 2 (diffuse atrophy), and 7 eyes (14.6%) did not have any myopic maculopathy lesions (category 0). None of the eyes had category 3 (patchy atrophy) or category 4 (macular atrophy) in both groups. In 31 eyes with the diffuse atrophy, 7 eyes with a staphyloma and 24 eyes without a staphyloma had PDCA. The incidence of myopic maculopathy lesions equal to or severer than category 2 was significantly more frequent in eyes with a staphyloma than in eyes without it (7/7 vs 24/48; *P* = 0.037).

**Table 1:** Clinical characteristics of the eyes with or without a staphyloma in children.

	Present	Absent	*P* Value
Number of Eyes (patients)	7 (5)	48 (28)	
Age (y/o), mean ± SD (range)	15.2 ± 3.4 (11 to 18)	12.1 ± 4.0 (6 to 19)	0.149
BCVA (logMAR), mean ± SD (range)	0.08 ± 0.28 (-0.08 to 0.70)	-0.03 ± 0.08 (-0.18 to 0.15)	0.375
Refractive Error (D), mean ± SD (range)	-12.1 ± 4.0 (-17.0 to -7.3)	-10.6 ± 2.7 (-17.5 to -6.8)	0.377
Axial Length (mm), mean ± SD (range)	28.9 ± 2.2 (26.8 to 32.6)	27.8 ± 1.4 (25.0 to 32.0)	0.275
Myopic Maculopathy Lesions†			
Category 0 (No Myopic Maculopathy Lesion) (eyes)	0 (0%)	7 (14.6%)	0.037^#^
Category 1 (Tessellated Fundus) (eyes)	0 (0%)	17 (35.4%)	
Category 2 (Diffuse Chorioretinal Atrophy) (eyes)	7 (100%)	24 (50.0%)	
Category 3 (Patchy Atrophy) (eyes)	0 (0%)	0 (0%)	
Category 4 (Macular Atrophy) (eyes)	0 (0%)	0 (0%)	
Category 2 to Category 4 (eyes)	7 (100%)	24 (50.0%)	
Parapapillary Diffuse Chorioretinal Atrophy (eyes)	7 (100%)	24 (50.0%)	0.037^#^

BCVA; Best Corrected Visual Acuity, y/o; years old, SD; standard deviation, D; diopter, N.S.; not significant

^#^Chi square test

^†^; myopic maculopathy lesions have been classified according to META-PM analyses

The retinal and choroidal thicknesses in the eyes with and without a staphyloma are shown in Table 2. The subfoveal choroid was significantly thinner in the eyes with a staphyloma than the eyes without a staphyloma. There were no significant differences in the subfoveal retinal thickness and in the choroidal thickness 2500 μm nasal to the fovea, 2500 μm temporal to the fovea, 2500 μm superior to the fovea, and 2500 μm inferior to the fovea between the 2 groups. The choroidal thickness 2000 μm nasal to the optic disc was significantly thinner in the eyes with a staphyloma (75.3 ± 26.2 μm) than those without a staphyloma (116.9 ± 45.8 μm; *P* = 0.015).

**Table 2:** Retinal and choroidal thickness of eyes with and without staphyloma in children.

	Staphyloma		
	Present	Absent	*P* Value
Number of Eyes (patients)	7 (5)	48 (28)	
Thickness of Retina and Choroid†			
Central Retina (μm), mean ± SD (range)	168.0 ± 31.7 μm (131 to 201)	189.8 ± 20.6 μm (146 to 230)	0.136
Subfoveal Choroid (μm), mean ± SD (range)	103.3 ± 79.9 μm (25 to 271)	151.3 ± 51.4 μm (45 to 297)	0.016*
Temporal Choroid (μm), mean ± SD (range)	163.9 ± 98.1 μm (63 to 363)	189.3 ± 53.3 μm (75 to 334)	0.114
Nasal Choroid (μm), mean ± SD (range)	68.6 ± 43.4 μm (16 to 158)	67.4 ± 35.9 μm (11 to 180)	0.990
Superior Choroid (μm), mean ± SD (range)	157.4 ± 92.6 μm (65 to 321)	191.4 ± 42.8 μm (115 to 314)	0.075
Inferior Choroid (μm), mean ± SD (range)	134.6 ± 55.1 μm (85 to 234)	170.4 ± 43.0 μm (46 to 270)	0.060
Choroid at 2000 μm Nasal from ON (μm), mean ± SD (range)	75.3 ± 26.2 μm (40 to 103)	116.9 ± 45.8 μm (48 to 270)	0.015*

ON; Optic Nerve, SD; standard deviation, D; diopter, N.S.; not significant

* Mann—Whitney U test

^†^; see Methods for the retinal thickness measurement

## Discussion

With the use of WF-OCT, the clear and objective detection of posterior staphylomas was possible in the current study. The results showed that among the 55 highly myopic eyes of 30 subjects, 7 eyes (12.7%) had a posterior staphyloma. Although staphylomas are generally considered to be pathological changes that develop in later life, the results showed that posterior staphylomas can be present at a much younger age than they had been believed.

All the 7 eyes with a staphyloma had a posterior displacement of the sclera in the staphylomatous area. Among the two OCT features of staphyloma edges reported by Shinohara et al., [8] the gradual thinning of the choroid from the periphery towards the edge of a staphyloma and a gradual re-thickening of the choroid in direction towards the posterior pole were observed in all of the eyes with a staphyloma. However, the inward protrusion of the sclera was found in only 2 of the 7 eyes. These results are different from the presence of 2 OCT features at staphyloma edges were consistently found in older subjects (100 eyes of 56 patients, mean age; 67.9 ± 10.7 years) with a staphyloma.[8] This difference suggests that the choroidal thickness changes toward and away from the staphyloma edge as well as the scleral posterior displacement in the staphylomatous area may develop first. Later, when the staphyloma becomes deeper, the inward protrusion of the sclera at the staphyloma edges might occur.

Statistical comparisons showed that the eyes with a staphyloma had diffuse chorioretinal atrophy (category 2) including PDCA significantly more frequently than eyes without a staphyloma. PDCA suggests a later development of pathologic myopia in adults as was suggested by Yokoi et al.[17] Considering that staphylomas are hallmark signs of pathologic myopia, these results suggest that PDCAs are also an important sign for the development of staphyloma in later life. The fact that one-half of the eyes without a staphyloma had PDCA also suggests that PDCA may precede the staphyloma formation.

The type of staphyloma was the peripapillary type in 4 of the 7 eyes, and the wide macular type in the remaining 3 eyes. Such high prevalence of peripapillary staphylomas is interesting, although the number of subjects was small. In earlier studies on adults by three-dimensional MRI, Ohno-Matsui [11] (mean age; 64.3 years) and Shinohara et al.[8] by WF-OCT and three-dimensional MRI (mean age; 67.9 years) reported that the wide macular type of staphyloma was the most predominant type in the older subjects. Although the detecting methodology was different, Curtin [1] also reported that wide macular type was the most frequent type based on stereoscopic fundus examinations. Interestingly, Ohno-Matsui et al.[19] reported a case of unilateral high myopia whose non-highly myopic eye had a peripapillary staphyloma and whose highly myopic eye had double staphylomas, a peripapillary staphyloma within a wide macular staphyloma. The results of these studies combined with the results of the current study suggest that a peripapillary staphyloma may develop prior to a macular staphyloma. It would be possible that staphylomas first develop around the optic nerve and later enlarges to involve the macula, or a macular staphyloma could develop independently in addition to pre-existing peripapillary staphyloma. However, without an accurate information on when staphylomas develop or without longitudinal follow-up data, it is difficult to conclude which type of staphyloma develops first.

The results showed that the differences in the subfoveal retinal thickness and choroidal thicknesses (superior, inferior, temporal, nasal) between the eyes with and without a staphyloma were not significant. The lack of significant differences in the choroidal thickness nasal to the fovea between the eyes with and without a staphyloma may seem contradictory to the earlier study by Yokoi et al.[18] Yokoi et al reported that myopic children with PDCA had significantly thinner choroid nasal to the fovea than control children in the Gobi Desert Children Eye Study. In contrast, the present study compared highly myopic children with and without a staphyloma. This may suggest that regardless of co-existing staphylomas, the choroid nasal to the fovea might already be thin in highly myopic children. To support this, the mean thickness of choroid nasal to the fovea was very thin in the eyes with and without a staphyloma (68.6 ± 43.4 μm and 67.4 ± 35.9 μm).

Interestingly, the subfoveal choroid and the choroid nasal to the optic disc were significantly thinner in the eyes with a staphyloma than in eyes without a staphyloma. This also suggested that the choroidal thinning did not occur uniformly within the posterior fundus in the eyes with a staphyloma, and the subfoveal choroid as well as the choroid nasal to the optic disc might become thinner than other regions. Such non-uniform choroidal thinning may cause great variations of the choroidal thickness which may predispose the scleral curvature changes. Another explanation would be that due to the high prevalence of peripapillary staphyloma, the subfovea and the area nasal to the optic disc might be more affected. Other regions, temporal, superior, and inferior to the fovea, are often outside the peripapillary staphyloma and thus these regions might be less affected.

There are several limitations in this study. First, this was not a longitudinal study and whether a gradual thinning of the choroid without the protrusion of the sclera finally becomes the typical edge accompanying scleral inward protrusion is not clear. Second, this study included highly myopic patients who attended a tertiary referral center. The results may therefore not represent the general population of highly myopic individuals. Third, we used the 12 radial scan images for the evaluation of the edge of a staphyloma or thinning of the choroid without a protrusion of the sclera. Therefore, it might be possible that the scleral or choroidal change was not detected if they existed between the scan lines. In spite of these limitations, we believe that our results showed the important features of the early signs of a staphyloma.

In conclusion, we detected and analyzed the OCT features of ‘early’ staphylomas in children and teenagers. The changes in the choroidal thickness toward the staphyloma edge were seen in all patients with a staphyloma, although the scleral protrusion at the staphyloma edge was seen in limited patients. The large variation in the choroidal thickness may be an early sign of a staphyloma. The results may provide important clues in assessing the indication of preventive therapies against staphyloma formation in the future.
